# Hirsutine, an Indole Alkaloid of *Uncaria rhynchophylla*, Inhibits Late Step in Dengue Virus Lifecycle

**DOI:** 10.3389/fmicb.2017.01674

**Published:** 2017-08-30

**Authors:** Takayuki Hishiki, Fumihiro Kato, Shigeru Tajima, Kazufumi Toume, Masahito Umezaki, Tomohiko Takasaki, Tomoyuki Miura

**Affiliations:** ^1^Laboratory of Primate Model, Institute for Frontier Life and Medical Sciences, Kyoto University Kyoto, Japan; ^2^Department of Virology 1, National Institute of Infectious Diseases Tokyo, Japan; ^3^Division of Pharmacognosy, Institute of Natural Medicine, University of Toyama Toyama, Japan; ^4^Division of Chemo-Bioinformatics, Institute of Natural Medicine, University of Toyama Toyama, Japan; ^5^Kanagawa Prefectural Institute of Public Health Kanagawa, Japan

**Keywords:** dengue virus, antiviral, crude drug, herbal medicine, alkaloid, hirsutine, focus assay, replicon

## Abstract

Dengue virus (DENV) is transmitted to humans by *Aedes* mosquitoes and is a public health issue worldwide. No antiviral drugs specific for treating dengue infection are currently available. To identify novel DENV inhibitors, we analyzed a library of 95 compounds and 120 extracts derived from crude drugs (herbal medicines). In the primary screening, A549 cells infected with DENV-1 were cultured in the presence of each compound and extract at a final concentration of 10 μM (compound) and 100 μg/mL (extract), and reduction of viral focus formation was assessed. Next, we eliminated compounds and extracts which were cytotoxic using the 3-(4,5-dimethylthiazol-2-yl)-2,5-diphenyltetrazolium bromide assay. Hirsutine, an indole alkaloid of *Uncaria rhynchophylla*, was identified as a potent anti-DENV compound exhibiting high efficacy and low cytotoxicity. Hirsutine showed antiviral activity against all DENV serotypes. Time-of-drug-addition and time-of-drug-elimination assays indicated that hirsutine inhibits the viral particle assembly, budding, or release step but not the viral translation and replication steps in the DENV lifecycle. A subgenomic replicon system was used to confirm that hirsutine does not restrict viral genome RNA replication. Hirsutine is a novel DENV inhibitor and potential candidate for treating dengue fever.

## Introduction

Dengue virus (DENV) causes dengue fever and dengue hemorrhagic fever which is transmitted to humans by *Aedes* mosquitoes (Gubler, 1998). Dengue virus is mainly found in tropical and sub-tropical areas, and is a public health problem across the globe (WHO, 2017). One report estimated that 390 million dengue infections with 96 million clinical manifestations occurred annually (Bhatt et al., 2013), while another estimated that 3.9 billion individuals in 128 countries are at risk of DENV infection (Brady et al., 2012).

Dengue virus belongs to the family Flaviviridae, which includes three genera, *Flavivirus*, *Pestivirus*, and *Hepacivirus*. Flaviviruses include the four serotypes of DENV (DENV-1–4), Zika virus, Japanese encephalitis virus, yellow fever virus, tick-borne encephalitis virus, and several other viruses. These are enveloped viruses approximately 50 nm in diameter, containing a single-stranded positive-sense RNA genome of approximately 11 kb (Lindenbach et al., 2007). A single long open-reading frame of the viral RNA encodes a polyprotein that is processed by viral and host-cellular proteases into three structural proteins [capsid (C), precursor membrane (prM), and envelope (E)] and seven non-structural (NS) proteins (NS1, NS2A, NS2B, NS3, NS4A, NS4B, and NS5). The structural proteins form the viral particles. Non-structural proteins are necessary for viral genome replication and evasion from host immune response (Chambers et al., 1990).

Antiviral agents target both viral factors and host-cellular factors. Multiple antiviral compounds have been identified using viral-enzyme activity assays, replicon assays, and DENV infection assays (Noble et al., 2010; Lim et al., 2013; Yang et al., 2014; Kato and Hishiki, 2016; Kato et al., 2016). Although many synthetic compounds and natural products inhibit DENV replication *in vitro* and *in vivo*, no antiviral drug has yet been approved for the treatment of DENV infection (Lim et al., 2013).

Many natural compounds have traditionally been applied to treat a variety of diseases, such as gastroenteritis, abdominal pain, jaundice, and diarrhea. Moreover, those also have been used for treatment of several viral infectious diseases (Li and Peng, 2013). To identify natural compounds which exhibit inhibitory effect on DENV propagation and clarify their antiviral mechanism, we performed a focus assay using compound and extract libraries derived from crude drug (herbal medicine). We identified hirsutine as an alkaloid displaying potent anti-DENV activity and low cytotoxicity. Our data suggest that hirsutine interferes with late step in DENV lifecycle.

## Materials and Methods

### Cells and Viruses

Human lung carcinoma cells (A549) and baby hamster kidney cells (BHK-21) were maintained in Eagle’s minimum essential medium (EMEM; Life Technologies, Carlsbad, CA, United States) supplemented with 10% fetal bovine serum (FBS; JR Scientific, Woodland, CA, United States), penicillin, and streptomycin sulfate (Nacalai Tesque, Kyoto, Japan) at 37°C with 5% CO_2_. Hybridoma-producing anti-E antibody (HB-112) was maintained in RPMI 1640 medium (Life Technologies, Carlsbad, CA, United States) supplemented with 10% FBS and antibiotics at 37°C with 5% CO_2_.

The viruses used in this study included DENV-1 (02-20 strain) (Tajima et al., 2006), DENV-2 (16681 strain), DENV-3 (09-59 strain), and DENV-4 (09-48 strain). The strains were cultured in Vero cells, and stored at -80°C. Dengue virus titer was determined by plaque assay as summarized below.

### Focus Assay and Herbal Medicine Library

The focus assay was performed as described previously (Kato et al., 2016). Briefly, A549 cells were seeded and infected with DENV-1 in a 96-well plate. One hour post-infection (hpi), EMEM containing 2% FBS, 1% methyl cellulose, and each tested compound or extract at a final concentration of 10 μM (compound), and 100 μg/mL (extract) was added. Three days post-infection, cells were incubated with anti-E antibody (4G2) as the primary antibody. Next, Dako Envision kit/HRP was used as the secondary antibody, and 3,3′-diaminobenzidine tetrahydrochloride was used for staining.

A herbal medicine library (INM deposited WAKANYAKU library, Institute of Natural Medicine, University of Toyama) used for screening in this study consist with the 120 extracts of crude drugs and the 95 compounds derived from crude drug. All crude drugs used in this study were purchased from Tochimoto Tenkaido (Osaka, Japan). The 50.0 g of each crude drug was extracted with 400 mL of distilled water by gently boiling for 60 min. The decoction was filtrated and freeze-dried to yield dry extract powder. Each crude drug extract was dissolved in ultra-pure water at a concentration of 10 mg/mL and kept at -80°C before use. The voucher specimen of these crude drugs and the voucher samples of these extracts were deposited in the Museum of Materia Medica, Institute of Natural Medicine (TMPW), University of Toyama, Japan. The crude drug derived compounds, purchased from WAKO (Osaka, Japan), Funakoshi (Tokyo, Japan), or Nagara Science (Gifu, Japan), were dissolved in dimethyl sulfoxide (DMSO) at a concentration of 10 mM and kept at -80°C before use.

### Cytotoxicity Assay

Cell viability was assessed by 3-(4,5-dimethylthiazol-2-yl)-2,5-diphenyltetrazolium bromide (MTT) assay. A549 cells were cultured in a 96-well plate with the selected compound (0–10 μM) or extract (0–100 μg/mL). After a 3-day incubation, 30 μL of 0.5% MTT solution was added to each well. After incubation for 3 h, the supernatant was replaced with 100 μL of methanol with 1% Triton X-100, and then vortexed to dissolve the formazan. Absorbance at 565 nm and reference at 655 nm were measured using a microplate reader (Berthold Technologies, Bad Wildbad, Germany). The half-maximal cytotoxicity concentration (CC_50_) was calculated using the Reed and Muench method (Reed and Muench, 1938).

### Plaque Assay

Baby hamster kidney-21 cells were cultured in a 12-well plate and infected with serial dilutions of virus suspension, then incubated at 37°C for 1 h. At 1 hpi, cells were incubated in EMEM containing 2% FBS and 1% methylcellulose. After 5 days of incubation, the cells were fixed with paraformaldehyde and stained with methylene blue. Virus titer was calculated as plaque-forming units (PFU)/mL. The half-maximal effective concentration (EC_50_) against each virus was calculated using the Reed and Muench method.

### Time-of-Drug-Addition Assay and Time-of-Drug-Elimination Assay

To conduct the time-of-drug-addition assay, A549 cells were cultured in a 24-well plate and infected with DENV-1 at a multiplicity of infection (MOI) of 1.0. Next, the cells were rinsed twice with phosphate-buffered saline, and fresh medium was added. At 0, 2, 4, 6, 8, 12, and 18 hpi, hirsutine at a final concentration of 10 μM was added to the infected cells. At 24 hpi, the cell culture supernatant was collected, and viral titer was assessed by plaque assay.

For the time-of-drug-elimination assay, A549 cells were cultured in a 24-well plate and infected with DENV-1 at MOI of 1.0, including hirsutine at a final concentration of 10 μM. Next, the cells were rinsed with phosphate-buffered saline twice, and fresh medium containing 10 μM hirsutine was added. At 2, 4, and 6 hpi, the cells were rinsed with phosphate-buffered saline twice and fresh medium was added. At 24 hpi, the cell culture supernatant was collected, and viral titer was measured by plaque assay.

### Transient Replicon Assay

A transient replicon assay was conducted using DGL2 and DGL2-mut as described previously (Kato et al., 2014). A549 cells were cultured in a 24-well plate and transfected with DGL2 and DGL2-mut using X-tremeGENE HP DNA transfection reagent (Roche, Basel, Switzerland); 10 μM of hirsutine was added at baseline. The cell culture medium was collected and replaced with fresh medium containing 10 μM of hirsutine every 24 h. The Gaussia luciferase activity in the culture medium was measured using a microplate reader.

### Western Blot Analysis

Western blotting was performed as described previously (Hishiki et al., 2014). Briefly, DENV-1-infected A549 cells were co-cultured with 10 μM hirsutine, 100 μM ribavirin, or 10 μM bromocriptine. Protein samples were separated by SDS-PAGE and transferred to polyvinylidene fluoride blotting membranes (GE Healthcare, Little Chalfont, United Kingdom). Anti-NS3 rabbit polyclonal antibodies (GTX124252; GeneTex, Irvine, CA, United States) and anti-glyceraldehyde-3-phosphate dehydrogenase (GAPDH) mouse monoclonal antibodies (MAB374; Millipore, Billerica, MA, United States) were used as primary antibodies.

## Results

### Identification of Inhibitors with Anti-DENV Activity from the Compound and Extract Libraries

**FIGURE 1:**
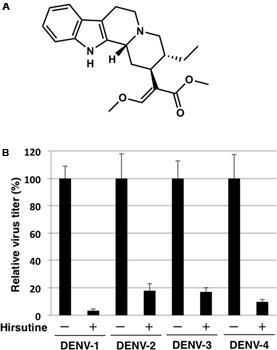
Hirsutine inhibits replication of dengue virus serotypes DENV-1–4. **(A)** Structural formula of hirsutine. **(B)** Hirsutine inhibits viral replication of all four serotypes. A549 cells were infected with DENV-1–4 at a multiplicity of infection (MOI) of 0.01 in the presence of 10 μM hirsutine. Cell culture supernatants were collected 72 hpi, and viral titers were determined by plaque assay. All data were normalized to their control (0 μM of hirsutine). Each data point represents the mean ± standard deviation from triplicate experiments.

To identify novel DENV inhibitors, we applied a focus reduction assay using a library of compounds and extracts derived from crude drug (herbal medicine) as a first screening. DENV-1 (02-20 strain)-infected A549 cells were treated with each compound and extract. Three days post-infection, the reduction in viral focus formation was assessed. Among 95 compounds and 120 extracts, 11 compounds and 9 extracts suppressed focus formation. Next, to exclude cytotoxic compounds and extracts, the MTT assay was conducted as a second screening. Furthermore, the suppressive effect of candidate inhibitors was also analyzed using a plaque assay as a third screening. Finally, the EC_50_ against DENV-1 and the CC_50_ were analyzed as a fourth screening. Two compounds (hirsutine and timosaponin A-III) and three extracts (Albizziae Cortex, Silktree Albizia Bark; Phellodendri Cortex, Phellodendron Bark; and Arecae Semen, Areca) were identified as potent inhibitors of DENV-1 (**Table 1**). Among the five candidate inhibitors, we focused on hirsutine (EC_50_, 1.97 μM; CC_50_ > 10 μM) because of studies reporting its antiviral activity against the influenza A virus (Takayama et al., 1997; Tietze and Zhou, 1999; **Table 1** and **Figure 1A**). Next, we analyzed the antiviral activity of hirsutine against other DENV serotypes using the plaque assay. The virus titer of all four DENV serotypes decreased by 10 μM hirsutine (**Figure 1B**). These results show that hirsutine inhibits viral replication of DENV-1–4.

**Table 1:** Anti-DENV activity of compounds and extracts derived from crude drugs.

Compound	EC_50_^a^ (μM)	CC_50_^b^ (μM)	SI^c^
Hirsutine	1.9	>10	>5.3
Timosaponin-AIII	1.7	>10	>5.9

**Extract**	**EC_50_^a^ (μg/mL)**	**CC_50_^b^ (μg/mL)**	**SI^c^**

Albizziae Cortex	2.9	>100	>34.5
Phellodendri Cortex	3.9	>100	>25.6
Arecae Semen	37.5	>100	>2.7


^a^*Half maximal effective concentration; EC_50_*. ^b^*Half maximal cytotoxicity concentration; CC_50_*. ^c^*Selectivity index; SI = CC_50_/EC_50_*.

### Hirsutine Does Not Inhibit the Entry and Release Stage of the DENV Lifecycle

**FIGURE 2:**
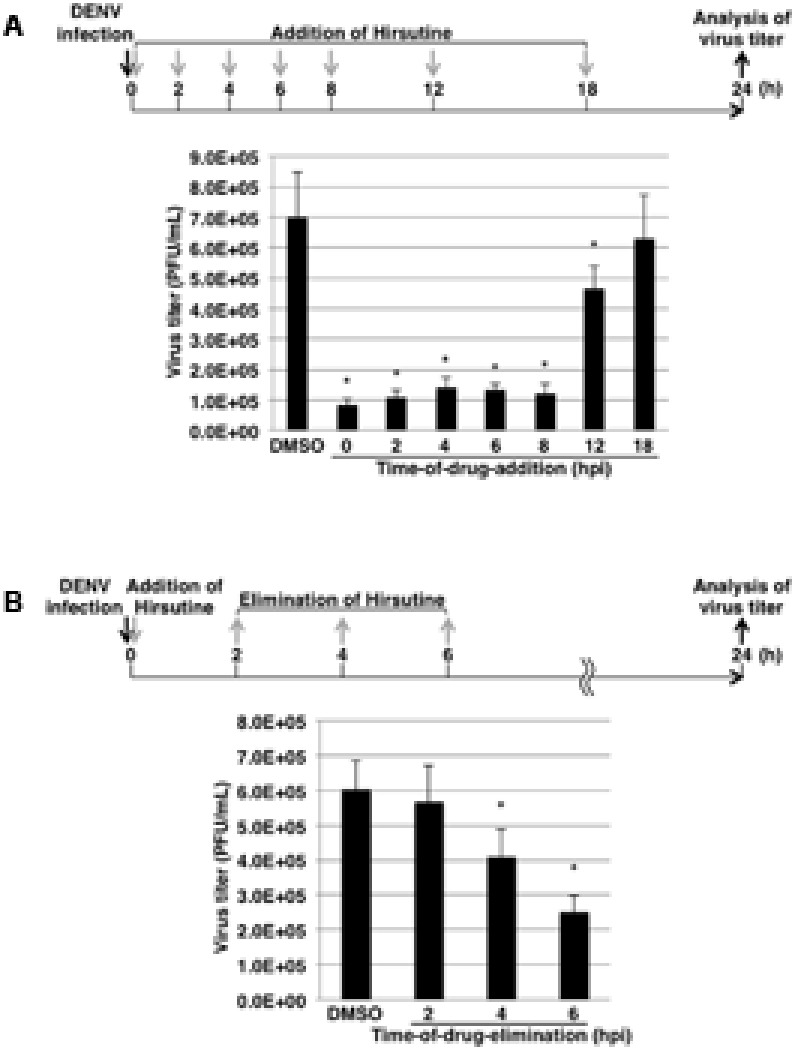
Hirsutine inhibits the late stage of the dengue virus lifecycle. **(A)** Time-of-drug-addition assay. Hirsutine (10 μM) was added at 0, 2, 4, 6, 8, 12, and 18 h post-DENV-1 infection. After 24 h, virus titers in the culture supernatant were analyzed by plaque assay. **(B)** Time-of-drug-elimination assay. Hirsutine (10 μM) was treated for 0–2, 0–4, and 0–6 h post-DENV-1 infection. After 24 h, virus titers in the culture supernatant were analyzed by plaque assay. ^∗^*p* < 0.05. Each data point represents the mean ± standard deviation from triplicate experiments. DMSO, dimethyl sulfoxide; hpi, hours post-infection; and PFU, plaque-forming units.

To clarify which stage of the DENV lifecycle is targeted by hirsutine, we conducted a time-of-drug-addition assay. At 0, 2, 4, 6, 8, 12, and 18 hpi, the culture medium was replaced by medium containing 10 μM hirsutine. At 24 hpi, the viral titer in the culture supernatant was assessed by plaque assay. As shown in **Figure 2A**, hirsutine reduced the virus titer significantly up to 12 hpi. This suggests that hirsutine inhibits the pre-release step of the viral particle in the DENV lifecycle. Next, to determine the target of hirsutine during the DENV replication stage, we conducted a time-of-drug-elimination assay. Hirsutine did not decrease the virus titer in the culture supernatant after a 2-h treatment, although the virus titer did decrease after a 4-h treatment (**Figure 2B**). Thus, we speculated that the viral attachment and entry stage was not the target of hirsutine.

### Hirsutine Does Not Suppress Viral-Genome RNA Translation and Synthesis

**FIGURE 3:**
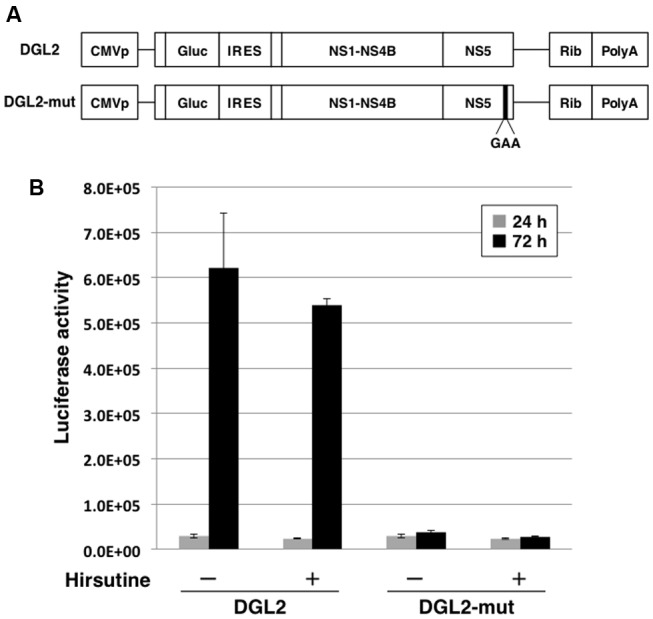
Hirsutine does not inhibit subgenomic reporter replicon activity. **(A)** Schematic representation of the replicon system, which uses DGL2 and DGL2-mut. DGL2-mut was inserted in non-replicative mutation into RNA-dependent RNA polymerase (RdRp). CMVp, cytomegalovirus promoter; Gluc, secretory Gaussia luciferase; IRES, internal ribosome entry site; GAA, mutation in the active center for RdRp; and Rib, ribozyme sequence. **(B)** Replicon plasmids DGL2 and DGL2-mut were transfected to A549 cells in the presence of 10 μM hirsutine. After 24 and 72 h, luciferase activity in the culture supernatant was analyzed. Each data point represents the mean ± standard deviation from triplicate experiments.

To further clarify the mechanism of hirsutine-mediated suppression of DENV replication, we performed a reporter subgenomic replicon assay. The transient replicon system is a useful tool for estimating the stage of action (**Figure 3A**; Kato et al., 2014). After 24 h and 72 h of replicon plasmid transfection, the Gaussia luciferase activity in the culture supernatant was analyzed. As shown in **Figure 3B**, hirsutine did not reduce luciferase activity levels 24 h and 72 h post-transfection with DGL2 and with non-replicate DGL2-mut. These results indicate that hirsutine did not inhibit DENV replication during viral-genome RNA translation and synthesis.

### Hirsutine Does Not Inhibit Viral-Genome RNA Replication

**FIGURE 4:**
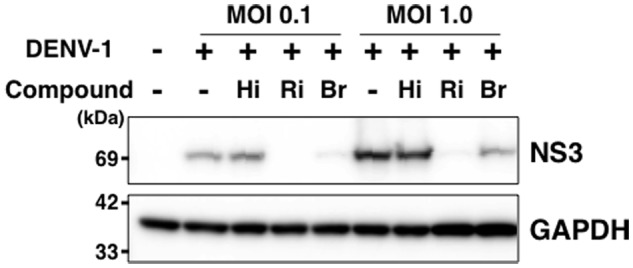
Hirsutine does not inhibit viral genome RNA replication. A549 cells were infected with DENV-1 at a MOI of 0.1 and 1.0 in the presence of 10 μM hirsutine (Hi), 100 μM ribavirin (Ri), or 10 μM bromocriptine (Br). Cell lysate was collected 18 h post-infection, and DENV-NS3 (top) and GAPDH (bottom) protein expression levels were assessed by Western blot.

Finally, we analyzed the expression level of DENV-NS3 protein with and without hirsutine, ribavirin, and bromocriptine during a single lifecycle. At 18 hpi in the presence or absence of 10 μM hirsutine, 100 μM ribavirin, or 10 μM bromocriptine, the cell lysate was collected and analyzed by Western blot. NS3 protein expression levels did not change in the presence of hirsutine, although they decreased significantly in the presence of ribavirin and bromocriptine (**Figure 4**), which are known inhibitors of DENV genome RNA replication (Kato et al., 2016). Collectively, these results suggest that hirsutine does not suppress DENV genome RNA replication, but inhibits the viral particle assembly, budding, or release step.

## Discussion

Dengue virus is the most common human arthropod-borne virus and is a major public health concern worldwide, mainly in tropical and sub-tropical regions. Although a tetravalent dengue vaccine based on a yellow fever backbone has been licensed in certain countries recently, its efficacy is not equal among the four serotypes. Moreover, no specific antiviral drugs against dengue infection are currently available; hence, development of antiviral agents is desirable.

In this study, we screened compound and extract libraries derived from crude drugs (herbal medicines) by using a focus assay to identify DENV inhibitors. The focus assay is not high-throughput, but permits the assessment of all DENV lifecycle steps. We used a combination of DENV-1 (02-20 strain) and A549 cells because of clear focus formation and easy-to-distinguish positive/negative signals in 96-well plates. Through screening, we identified a novel DENV inhibitor, hirsutine, in the herbal medicine library (WAKANYAKU library).

Hirsutine is one of the major indole alkaloids of *Uncaria rhynchophylla* and has been reported to possess anti-hypertensive, anti-arrhythmic, cardioprotective, and anti-metastatic properties through its effects on inhibition of Ca^2+^ influx and the release of intracellular Ca^2+^ (Horie et al., 1992; Wu et al., 2011; Lou et al., 2014). Moreover, calcium homeostasis has been reported to associate with disease severity of dengue (Shivanthan and Rajapakse, 2014). Thus, it is possible that antiviral activity of hirsutine may be related to calcium homeostasis. Furthermore, hirsutine was found to exhibit inhibitory effects against the influenza A virus (subtype H3N2), the EC_50_ of hirsutine was over 10-fold more effective than ribavirin, although the mechanism has not been clarified yet (Takayama et al., 1997; Tietze and Zhou, 1999). Therefore, based on these reports and our findings in this study, hirsutine may be effective against broad viral replication.

Multiple natural products from plants have been reported to display potent anti-DENV replication activity, and some were shown to target the activity of viral enzymes such as NS3 protease and NS5 polymerase (Jassim and Naji, 2003; Kiat et al., 2006; Allard et al., 2011; Oliveira et al., 2017). However, our results from a subgenomic replicon assay and Western blot analysis indicate that hirsutine is not involved in viral enzyme activity. Other viral proteins or host factors are likely to be involved in the antiviral mechanism of hirsutine. Our results suggest that hirsutine suppresses late step of DENV. Several studies have indicated that viral proteins and host factors are associated with DENV particle assembly, budding, or release step (Murray et al., 2008; Fernandez-Garcia et al., 2009; Apte-Sengupta et al., 2014). Viral particle assembly occurs within compartment of endoplasmic reticulum (ER) vesicles, and particles then released from cells through the host cell secretory machinery. These steps of DENV lifecycle require complex molecular interaction between viral and host cell factors. Therefore, it is possible that hirsutine may influence these factors.

Time-of-drug-addition and time-of-drug-elimination assays suggested that hirsutine inhibited not only the viral entry step or the viral genome RNA replication step but also the viral particle assembly, budding, or release step in the DENV lifecycle. Furthermore, the replicon assay showed that hirsutine did not restrict the viral genome translation and replication steps. Because our subgenome replicon, DGL2, deleted almost the entire structural region of DENV-1, the assay could specifically analyze the viral genome translation and replication steps (Kato et al., 2014). Hence, the replicon system is a useful tool for screening antiviral reagents and for elucidating the mechanism of action in the virus lifecycle (Kato and Hishiki, 2016).

We also found anti-DENV activity in the extracts of crude drugs, Albizziae Cortex (EC_50_, 2.9 μg/mL; CC_50_, >100 μg/mL), Phellodendri Cortex (EC_50_ 3.9 μg/mL; CC_50_, >100 μg/mL), and Arecae Semen (EC_50_, 37.5 μg/mL; CC_50_, >100 μg/mL). In near future, we will clarify which component of these extracts contributes to antivirus activity. The extract of Phellodendri Cortex has been reported to affect the influenza A virus, vesicular stomatitis virus, Newcastle disease virus, and herpes simplex virus, by inducing type I interferon or pro-inflammatory cytokines (Kim et al., 2016). Therefore, the anti-DENV replication activity of Phellodendri Cortex may have a similar mechanism.

Taken together, our findings provide strong support for the use of these compounds and extracts to treat DENV infections. We found that hirsutine exerts antiviral activity against all DENV serotypes, and may restrict late step in the DENV lifecycle. However, the detailed mechanism underlying antiviral activity must be identified before an anti-DENV drug can be developed. Further studies are ongoing to elucidate the mechanism of antiviral activity of hirsutine.

## Author Contributions

TH and FK performed the experiments and analyzed the data; TH wrote the manuscript; ST, KT, MU, TT, and TM provided reagents; and FK, ST, KT, TT, and TM interpreted data and edited the manuscript.

## Conflict of Interest Statement

The authors declare that the research was conducted in the absence of any commercial or financial relationships that could be construed as a potential conflict of interest.
